# The Southern Surgical and Gynecological Society

**Published:** 1890-12

**Authors:** 


THE Southern Surgical and Gyecological Association held a most interesting and profitable meeting at Atlanta, Ga., on Tuesday, Wednesday, and Thursday, November 11, 12, and 13, 1890. The papers read and the discussions thereon were, for the most part, of a high order of merit, and the scientific work done during the three days' session will fill a volume that any medical body might be proud to produce. The medical profession of Atlanta did all that could be done to make the sojourn of the visitors pleasant, and the social entertainments were of a delightful character. The Capital City Club opened its doors with a lavish hospitality, that could not be excelled in any city of the land, and the reception it gave the association on Wednesday evening, November 12th, will be remembered a long time as a conspicuous gathering of beautiful women and renowned men, in which music, flowers, wine, and substantial viands blended in a most artistic and gratifying manner. The next meeting will be held in Richmond, Va., in November, 1891, when Dr. Hunter McGuire will serve as Chairman of the Committee of Arrangements. The officers chosen for the ensuing year are: President, Dr. Lewis S. McMurtry, of Louisville; Vice-Presidents, Dr. J. McF. Gastin, of Atlanta, and Dr. J. T. Wilson, of Sherman, Texas; Secretary, Dr. W. E. B. Davis, of Birmingham, Ala.; Treasurer, Dr. H. P. Cochrane, also of Birmingham. The Judicial Council remains the same as last year, except that Dr. George J. Engelmann, of St. Louis, was chosen to fill the vacancy occasioned by the expiration of the term of Dr. Bedford Brown, of Alexandria, Va. The accomplished Secretary, Dr. W. E. B. Davis, deserves great praise for the excellent manner in which he conducted the affairs of the Association, and his continuance in office is an indication of what the future will vouchsafe to this able and scientific body of professional workers.

